# The Sulphur Response in Wheat Grain and Its Implications for Acrylamide Formation and Food Safety

**DOI:** 10.3390/ijms21113876

**Published:** 2020-05-29

**Authors:** Sarah Raffan, Joseph Oddy, Nigel G. Halford

**Affiliations:** Plant Sciences Department, Rothamsted Research, Harpenden AL5 2JQ, UK; sarah.raffan@rothamsted.ac.uk (S.R.); joe.oddy@tothamsted.ac.uk (J.O.)

**Keywords:** wheat, *Triticum aestivum*, asparagine metabolism, Maillard reaction, acrylamide, food safety, amino acid metabolism, glutathione, starch synthesis, sulphur deficiency

## Abstract

Free (soluble, non-protein) asparagine concentration can increase many-fold in wheat grain in response to sulphur deficiency. This exacerbates a major food safety and regulatory compliance problem for the food industry because free asparagine may be converted to the carcinogenic contaminant, acrylamide, during baking and processing. Here, we describe the predominant route for the conversion of asparagine to acrylamide in the Maillard reaction. The effect of sulphur deficiency and its interaction with nitrogen availability is reviewed, and we reiterate our advice that sulphur should be applied to wheat being grown for human consumption at a rate of 20 kg per hectare. We describe the genetic control of free asparagine accumulation, including genes that encode metabolic enzymes (asparagine synthetase, glutamine synthetase, glutamate synthetase, and asparaginase), regulatory protein kinases (sucrose nonfermenting-1 (SNF1)-related protein kinase-1 (SnRK1) and general control nonderepressible-2 (GCN2)), and basic leucine zipper (bZIP) transcription factors, and how this genetic control responds to sulphur, highlighting the importance of asparagine synthetase-2 (*ASN2*) expression in the embryo. We show that expression of glutamate-cysteine ligase is reduced in response to sulphur deficiency, probably compromising glutathione synthesis. Finally, we describe unexpected effects of sulphur deficiency on carbon metabolism in the endosperm, with large increases in expression of sucrose synthase-2 (*SuSy2*) and starch synthases.

## 1. Introduction

Sulphur nutrition has been known to affect cereal crop yield and grain quality for many years. Work on barley in the 1980s, for example, showed that sulphur deficiency caused an accumulation of free amino acids in the grain, with free (soluble, non-protein) asparagine levels increasing markedly [1]. That was considered to be of mainly academic interest at the time, and in the 1990s attention switched to the effect of sulphur nutrition on protein content and quality. Nitrogen fertilizer, of course, is required for farmers to achieve optimum yield and protein content in wheat, with UK farmers applying 250–300 kg of nitrogen per hectare to breadmaking wheat. This level of nitrogen is required in order to achieve the 13% protein content required for the Chorleywood Breadmaking Process, which is the most widely used method for breadmaking in the UK. However, sulphur nutrition is also important because sulphur deficiency leads to low levels of some classes of seed storage proteins, with negative effects on breadmaking quality. Zhao et al. [2], for example, showed decreased grain size in wheat under sulphur-limiting conditions, reduced yield, and increased synthesis of sulphur-poor storage proteins such as ω-gliadins and high molecular weight (HMW) subunits of glutenin at the expense of sulphur-rich proteins. Sulphur deficiency also reduced the size of the polymers that formed from these proteins.

The authors of that study noted that sulphur deficiency had become much more widespread in agricultural land in western Europe towards the end of the 20th Century. This was caused in part, ironically, by the huge decrease in atmospheric sulphur deposition that had been achieved in that period, with the switch to low-sulphur fuels such as natural gas and the fitting of coal- and oil-burning power stations with pre- and post-combustion systems for removing sulphur. Higher-yielding crops were also denuding soils of minerals more rapidly. The impact these factors had on soil sulphur levels was compounded by farmers using more ammonium nitrate-based fertilizers in preference to ammonium sulphate or superphosphate (a mixture of CaSO_4_·H_2_O and Ca(H_2_PO_4_)_2_·H_2_O), which contain less nitrogen but also provide sulphur. The authors also gave a sulphur requirement of wheat of about 15–20 kg per ha for optimum growth, grain yield, and quality [2], something that we will return to later in this review. Sulphur deficiency was subsequently reported to be affecting wheat production in Australia, New Zealand, and northern and western Europe, as well as the UK [3,4]. Soon it became clear that sulphur deficiency during wheat cultivation was also a problem affecting food safety; a realization that began with the discovery of acrylamide in common foods. In this review, we provide a brief history of the discovery of acrylamide in food and the response of regulatory authorities, describe how acrylamide forms from free asparagine and reducing sugars, and discuss the relationship between sulphur deficiency, the nitrogen-sulphur balance and free asparagine concentration in wheat grain. We describe the genetic control of asparagine synthesis and breakdown in wheat grain and how that control responds to sulphur, including the signalling pathways that are beginning to be elucidated. Finally, we describe evidence that sulphur deficiency has unexpected but substantial effects on genes involved in the regulation of carbon metabolism and starch synthesis.

## 2. The Discovery of Acrylamide in Food

**Figure 1:**
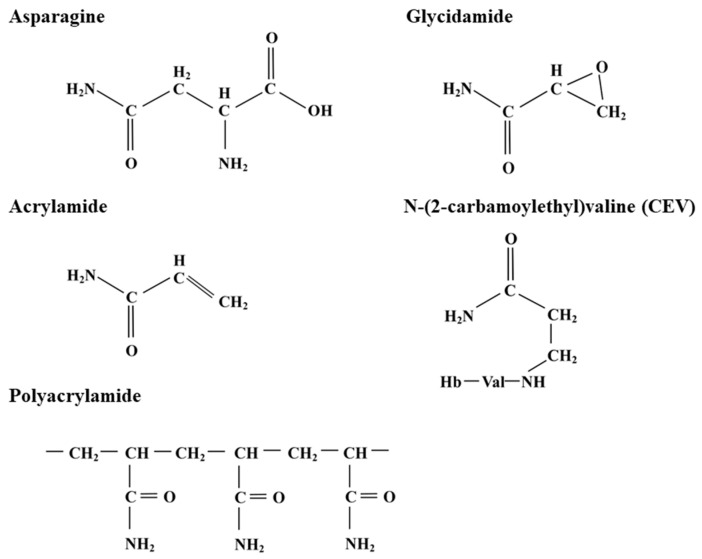
Diagrams showing the structure of asparagine; acrylamide, the carbon skeleton of which is derived entirely from asparagine; acrylamide chains in polyacrylamide, in which cross-links form between the nitrogen atoms on different chains to produce an insoluble matrix; glycidamide, the major metabolite of acrylamide; and the adduct N-(2-carbamoylethyl)valine (CEV), which forms through the reaction of acrylamide with the N-terminal valine residue of a globin chain of haemoglobin (Hb).

In 2002, it was reported that acrylamide (C_3_H_5_NO) (Figure 1) had been detected in a variety of popular foods [5]. Acrylamide is classified as an extremely hazardous substance in the USA and a serious health hazard with acute toxicity in the European Union. It is a potent neurotoxin, affects male reproduction, causes birth defects and is carcinogenic in laboratory animals [6,7]. It is also classed as a Group 2A carcinogen (probably carcinogenic to humans) by the International Agency for Research on Cancer (IARC) [8].

In its polymeric form (Figure 1), which is not considered to be toxic, acrylamide is used as a flocculant in wastewater and sewage treatment, and has a variety of other industrial uses. It is also a familiar laboratory chemical, being used, for example, for polyacrylamide gel electrophoresis (PAGE). The polymer may contain a small concentration of monomeric acrylamide as an impurity, making monomeric acrylamide a potential water pollutant, with a guideline value for its presence in drinking water set by the World Health Organisation at 0.5 µg per litre.

The discovery that acrylamide was present in food arose from studies on the effects of exposure to acrylamide in workers involved in acrylamide manufacture, and in smokers, since acrylamide is also present in tobacco smoke [9,10]. Both acrylamide and its metabolic derivative, glycidamide (C_3_H_5_NO_2_) (Figure 1), form adducts with haemoglobin in the blood, acrylamide reacting with the N-terminal valine residues of globin chains to produce N-(2-carbamoylethyl)valine (CEV) (Figure 1). The ratio of adducts to globin chains provides a quantitative measure of acrylamide exposure. It was noted in these studies that there were higher than expected levels of adducts in the control groups, leading to a search for the source of this acrylamide.

That search led to the discovery that acrylamide formed during the frying of animal feed [11], then to the demonstration that it formed during the cooking of common foods and was, therefore, present in the human diet [5]. Acrylamide can be classified as a processing contaminant, defined as a substance that is produced in a food when it is cooked or processed, is not present or is present at much lower concentrations in the raw, unprocessed food, and is undesirable either because it has adverse effects on product quality or because it is potentially harmful [12]. It does not form to detectable levels in any boiled foods, but is associated predominantly with fried, baked, roasted, or toasted potato and cereal products, as well as coffee (see [13] for review). Fried sweet potato and other storage root products may also be major contributors in some countries.

The discovery of acrylamide in common foods has presented food safety authorities with a difficult problem. Ideally, a toxic compound such as acrylamide should not be present in food at all, yet acrylamide forms from naturally-present precursors (Section 3) during every-day cooking and processing, and the levels typically found in some foods are many times higher than the tolerance level set for drinking water of 0.5 µg per litre. An analysis of manufacturers’ data on potato crisps (called chips in the USA) in Europe, for example, found that mean levels in 2002 were 763 µg per kg and by 2016, after 14 years of developing measures for reducing acrylamide formation, the levels were still at 412 µg per kg [14]. While this represented a large reduction, it was still uncomfortably high, and levels had plateaued between 2011 and 2016, suggesting that the easy gains in reducing acrylamide levels had already been made [14].

The European Commission has led the way in developing a regulatory system for acrylamide levels in food, beginning by monitoring levels of acrylamide in foods across Europe from 2003 onwards (see [13] for a comprehensive review). The current European Union (EU) Regulation on acrylamide in food is Commission Regulation (EU) 2017/2158 [15], which came into force on 11^th^ April 2018. This regulation includes the statement that “acrylamide in food potentially increases the risk of developing cancer for consumers in all age groups”. It sets Benchmark Levels for different food types, describing Benchmark Levels as performance indicators for the success of mitigation measures. It also includes detailed and compulsory mitigation measures to be adopted by all food businesses, and an explicit threat to impose Maximum Levels (i.e., levels above which it would be illegal to sell a product) in the future. Compliance with this and future regulations on acrylamide in food is one of the most difficult challenges facing the food industry in the EU, and other regulatory authorities around the world are likely to follow the EU’s lead.

## 3. Free Asparagine and Acrylamide Formation: The Maillard Reaction

Not long after publication of the report that acrylamide was present in common foods [5], it was shown that acrylamide could form from reducing sugars and free (soluble, non-protein) asparagine (C_4_H_8_N_2_O_3_), within the Maillard reaction [16,17,18,19]. In fact, the carbon skeleton of the acrylamide that forms is derived entirely from free asparagine (Figure 1), and thus free asparagine should be regarded as the true precursor for acrylamide formation, although it is convenient to consider reducing sugars as precursors as well. The predominant reducing sugars in plant tissues are glucose, fructose, and maltose, of which glucose and maltose are aldoses, with a highly reactive free aldehyde group when in the linear chain form, while fructose is a ketose, with a free keto group. Sucrose, the major transported sugar and the predominant simple sugar in most plant tissues is not a reducing sugar.

**Figure 2:**
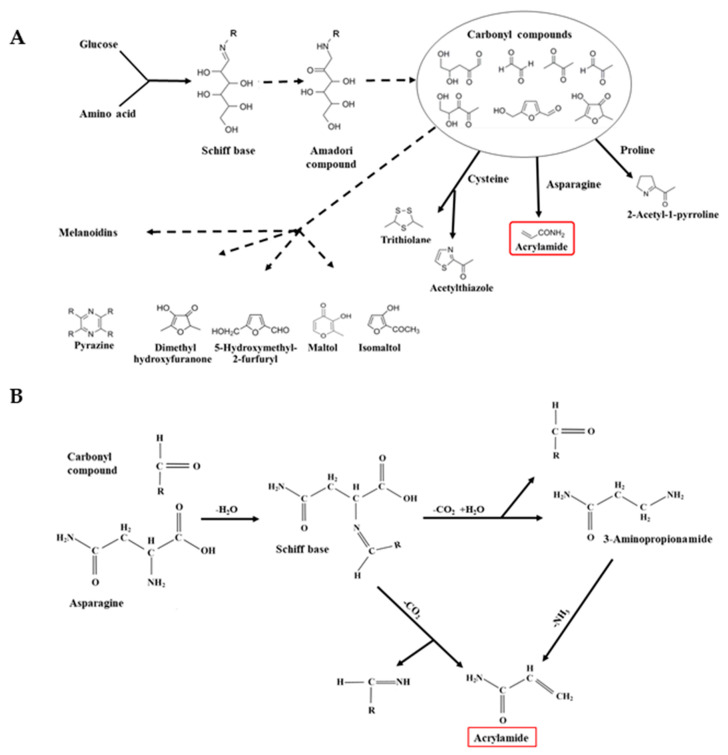
(**A**) Simple representation of the Maillard reaction, showing the Schiff base and Amadori compound formed from the reaction of glucose (an aldose) with free amino acids (fructose and other ketoses would form related Heynes compounds). Carbonyl compounds such as deoxyosones are formed by enolisation, deamination, dehydration, and fragmentation of Amadori and Heynes compounds, and react again with free amino acids to give a plethora of products, some of which are shown. (**B**) Formation of acrylamide by Strecker degradation of free asparagine [19].

The Maillard reaction was first described by a French chemist, Louis Camille Maillard, in 1912 [20], although the steps in the reaction as they are understood today were first proposed by an American chemist, John Hodge, as the “Hodge Scheme” in 1953 [21]. It comprises a series of non-enzymatic reactions between reducing sugars and amino groups, principally those of free amino acids. It is promoted by high temperature and low moisture content, and its products include melanoidin pigments, which are responsible for the brown colour in fried, baked, and roasted foods, and complex mixtures of compounds that impart flavour and aroma, including heterocyclic compounds such as pyrazines, pyrroles, furans, oxazoles, thiazoles, and thiophenes (Figure 2A). It is, therefore, responsible for the colours, flavours, and aromas that consumers expect and demand in fried, baked, roasted, and toasted foods, and for the specific characteristics that define food types and brands. This makes the acrylamide issue more difficult for food businesses to deal with, because any steps they take to reduce acrylamide formation are also likely to affect the levels of desirable Maillard reaction compounds in their products.

The reaction begins with the condensation of the carbonyl (C=O) group of a reducing sugar with an amino group, producing a Schiff base (Figure 2A) (an imine in which the nitrogen atom of the C=N group is attached to an organic group). If the sugar is an aldose, the Schiff base cyclises to give an N-substituted aldosylamine, such as glucosylamine from glucose (Figure 2A). Acid-catalysed rearrangement of the aldosylamine gives a 1,2-enaminol, which is in equilibrium with an N–substituted 1-amino-2-deoxyketose—these are known as Amadori rearrangement products (Figure 2A). Ketoses, such as fructose, give related Heyns rearrangement products.

Amadori and Heyns rearrangement products undergo enolisation, deamination, dehydration, and fragmentation, resulting in the production of compounds containing one or more carbonyl (C=O) groups, including deoxyosones, heterocyclic furfurals, furanones, and pyranones (Figure 2A). These highly reactive carbonyl compounds undergo further condensation reactions with other free amino acids and amines. One such reaction is Strecker degradation, which involves the deamination and decarboxylation of an amino acid to give an aldehyde, an α-aminoketone and carbon dioxide. The Strecker aldehydes and aminoketones that are produced include ethanal (fruity and sweet aroma and taste), methylpropanal (malty aroma and taste), and 2-phenylethanal (flower and honey aroma and taste). These compounds can contribute substantially to the flavour and aroma of the food and are, therefore, highly desirable. However, Strecker-type degradation of asparagine produces acrylamide (Figure 2B), which is definitely not desirable. The asparagine reacts with a carbonyl compound to produce a Schiff base. This is then converted to acrylamide by decarboxylation followed by either the removal of a substituted imine, or the elimination of a carbonyl group to produce an intermediate, 3-aminopropionamide, which is then converted to acrylamide by the removal of ammonia (Figure 2B) [19]. This makes 3-aminopropionamide an important intermediate in acrylamide formation [22].

The Maillard reaction is complex and multistep, and the relationship between precursor concentration and different products is not a simple one. Indeed, the relative importance of free asparagine and reducing sugar concentrations in determining acrylamide-forming potential varies between different crop products (see [13] for review). However, for wheat and rye grain and probably for other cereal grains as well, it is the concentration of free asparagine that determines acrylamide-forming potential (see [13] for review). The amount of acrylamide that actually forms in a product will depend on other factors as well, such as pretreatment to remove free asparagine, reducing cooking time and/or temperature, changes to the recipe, or other measures affecting cooking and processing. These approaches to the problem are not reviewed here, but have been compiled in a “Toolbox” by Food Drink Europe [23].

The Maillard reaction is not the only route proposed for acrylamide formation to occur, and more than one route may operate. For example, acrylamide has been shown to form in dry-heated wheat gluten extracts after the soluble components, which would include free amino acids and simple sugars, have been removed [24]. It has also been found in dried fruit, such as prunes and dates, the drying systems for which are typically around 60 °C or lower, which would be too low for the Maillard reaction to proceed. Nevertheless, the Maillard reaction route appears to be the predominant one in most products (see [13] for review).

## 4. The Effect of Sulphur Deficiency on Free Asparagine Concentrations in Wheat Grain

The discovery of acrylamide in food and the identification of free asparagine as its precursor made the understanding of factors that affect free asparagine concentration in wheat and other cereal grains important. This in turn led to renewed interest in the effect of sulphur nutrition on the concentration of free asparagine in wheat and other cereal grains, given that, as we have stated, sulphur deficiency was already known to increase free asparagine concentration in barley, at least in glasshouse experiments [1]. Measurements of free asparagine concentrations in wholemeal flour from three varieties of winter wheat grown in vermiculite in pots under glass showed dramatic increases of up to 30-fold in response to sulphur deficiency (from 5 mmol per kg to 153 mmol per kg in one variety) [25]. The concentrations of other free amino acids were also higher in the sulphur-deprived wheat, with glutamine, for example, increasing in one variety from 0.43 mmol per kg to 69 mmol per kg [25]. Vermiculite does not retain nutrients, and thus the wheat in those experiments was entirely dependent on nutrients supplied through watering. This meant that a very severe sulphur deficiency could be imposed. However, the same study also showed dramatic effects of sulphur supply on free asparagine concentration in flour from wheat grown in field plots treated with 0, 10, or 40 kg sulphur per hectare [25]. Grain from the plots with no added sulphur had an average free asparagine content of 66 mmol per kg compared with 3.7 mmol per kg in the grain from wheat grown with sulphur supplied at 40 kg per hectare. Even flour from the wheat grown with the addition of 10 kg sulphur per hectare had more than double the concentration of free asparagine and produced 58% more acrylamide on heating than flour from the wheat grown with 40 kg sulphur per hectare.

**Figure 3:**
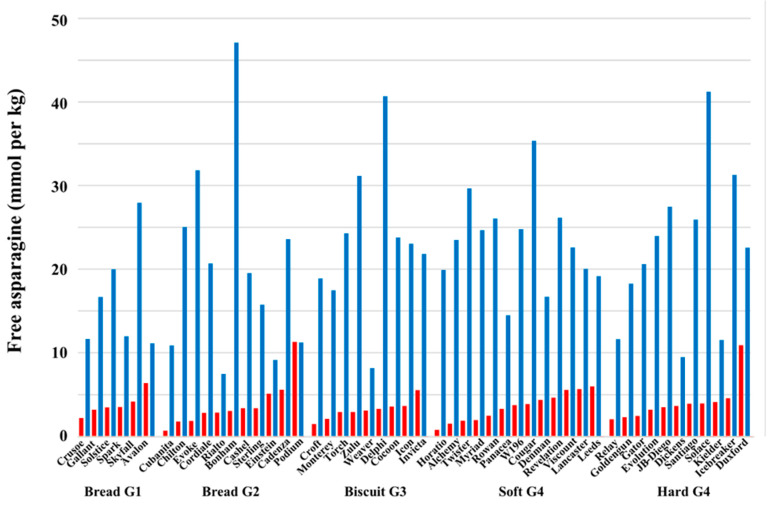
Mean free asparagine concentration in the grain of 50 varieties of winter wheat grown in a field trial in the UK in 2012-2013. Data are shown from split-plots in which wheat in half the plot was supplied with nitrogen and sulphur (red columns), while the wheat in the other half was supplied with nitrogen but not sulphur (blue columns). The varieties are shown separated into milling types. The effect of sulphur supply was significant (*p* = 0.007). Plotted from data provided in [29].

Subsequent studies have shown similar results [26,27,28,29,30]. Figure 3, for example, shows the free asparagine concentration in grain of 50 different varieties of winter wheat grown in a field trial in the UK in 2012-2013 [29]. The data were obtained from split-plots in which wheat in half the plot was supplied with nitrogen and sulphur (red columns), while the wheat in the other half was supplied with nitrogen but not sulphur (blue columns). The varieties are shown separated into milling types, using the National Association of British and Irish Millers (NABIM) classification [31]: Group 1, consistent milling and baking performance; Group 2, bread-making potential but not suited to all grists; Group 3, soft varieties used for biscuits, breakfast cereals, cakes, and similar products; Group 4, sub-grouped into hard and soft types, used mainly for animal feed and bioethanol, but incorporated into some grists for food use. Statistical analysis of all the data from the trials suggested that Group 4 (soft) wheats were most affected by sulphur deprivation [29]; however, individual varieties within each type responded differently to the treatment. One trend that did emerge was that varieties with low free asparagine in the sulphur-supplied condition were generally more affected by sulphur deprivation. Conversely, variety Podium had one of the highest concentrations of free asparagine in the sulphur-fed condition but showed no increase at all in the sulphur-deficient condition. This means that attempts to rank varieties for free asparagine concentration will be confounded if the varieties are not grown with sufficient sulphur.

These studies all analysed wholemeal rather than white flour. This is because the bran fractions of milled wheat grain contain higher concentrations of free asparagine than the white flour fraction [32]. This presents a dilemma with respect to consumer advice: the consumption of wholegrain cereal products has been shown to be beneficial to health (see [33] for review), so how should that be balanced against a still unknown risk of the higher acrylamide levels in wholegrain wheat products?

## 5. Interacting Effects of Sulphur and Nitrogen Fertilization on Free Asparagine Concentrations in Wheat Grain

Asparagine (Figure 1) contains no sulphur, yet it is much more responsive to sulphur supply in wheat grain than the amino acids that that do contain sulphur; i.e., methionine and cysteine [25,26,27,28,29]. Nitrogen has the opposite effect to sulphur, with increased nitrogen availability leading to more free asparagine accumulating in the grain [30,34,35,36]. One explanation for the increase in free asparagine in wheat grain in response to sulphur deficiency is that wheat uses free asparagine as a nitrogen store when it has insufficient sulphur to make sulphur-rich seed storage proteins [2,37]. If more nitrogen were available then more would have to be stored in this way. If that is the case then the balance of nitrogen and sulphur availability becomes important. Note that this is not a passive process but an active response by the plant (Section 6).

The importance of a balance between nitrogen and other nutrients on asparagine levels has also been discussed by Beato et al. [38], in which the authors propose a model to explain asparagine accumulation in tobacco roots during boron deficiency. In this model, boron deficiency increases proteolysis and reduces cellular hexose, thereby creating a high ammonia to hexose ratio. This increases the activity of glutamate dehydrogenase (GDH), which produces 2-oxoglutarate and ammonia. 2-Oxoglutarate is then fed into the tricarboxylic acid cycle while the ammonia is re-assimilated into asparagine via the glutamine synthetase (GS)–glutamate synthase (GOGAT) cycle and asparagine synthetase (ASN), allowing efficient recycling of nitrogen and carbon and the simultaneous detoxification of ammonia. As outlined above, asparagine levels in wheat increase with greater nitrogen availability, which is often available to the plant as ammonia. It is also clear that, in Arabidopsis, sulphur deficiency can downregulate glucose metabolism to cause a reduction in cellular hexose levels [39]. Consequently, the ratio of ammonia to hexoses in the cell may be a driving force behind asparagine accumulation and the molecular basis through which nitrogen, sulphur, and other nutrients control asparagine levels. However, this model has yet to be experimentally verified in wheat.

The uptake of many nutrients in wheat is also controlled in part by arbuscular mycorrhizal fungi (AMF). AMF are important for providing nutrients such as nitrogen and phosphorus to plants in return for carbon, and are known to impact asparagine levels in other species. For example, AMF colonisation increases levels of asparagine in sorghum shoots [40], tomato fruit [41] and Arabidopsis roots [42]. During nitrogen deprivation, however, levels of asparagine are decreased in more heavily colonised winter wheat roots [43], likely as a result of increased competition for nitrogen. The results of these studies show that AMF colonisation, like nitrogen fertilisation, can increase asparagine levels when nitrogen is abundant. The role of AMF in plant sulphur uptake is less well characterised, but evidence suggests that an increase in AMF root colonisation is likely to increase plant sulphate uptake. This could be achieved as a result of direct transport of AMF-scavenged sulphate to plants, upregulation of sulphate transporters in plant roots, or an increased association with bacteria that mobilise organic sulphur [44]. Consequently, AMF colonisation may reduce levels of asparagine by providing plants with sulphur and other nutrients; however, this effect is likely counterbalanced by the increased availability of nitrogen provided by AMF. It is currently unclear how different application rates of nitrogen and sulphur interact to impact AMF colonisation, but their application is likely to modulate whatever effect AMF may have on asparagine levels.

The UK’s Agriculture and Horticulture Development Board now advises farmers to apply sulphur to wheat at a rate of 10–20 kg sulphur per hectare to ensure that free asparagine concentrations are kept as low as possible [45]. This is equivalent to 25–50 kg per hectare SO_3_ (the standard unit used by the UK fertilizer industry). Our recommendation is that the higher level (20 kg sulphur per hectare) should be applied. We also advise that nitrogen fertilizer should not be applied unless it is accompanied with sulphur, and this has been standard practice in Sweden, for example, since the late 1990s. Ensuring that “good agricultural practices” are followed on fertilization, particularly to “maintain balanced sulphur levels in the soil and to ensure correct nitrogen application” is also included as a compulsory mitigation measure in European Commission Regulation (EU) 2017/2158 [15].

## 6. Genetic Control of Free Asparagine Concentration in Wheat Grain and its Response to Sulphur

**Figure 4:**
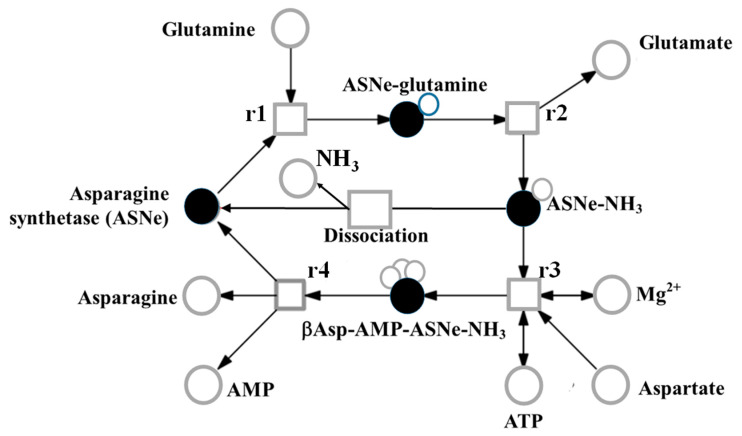
Model representing the reaction catalysed by asparagine synthetase, assuming mass action kinetics. The model comprises metabolites (open circles), the asparagine synthetase enzyme (closed circles), and reactions (squares), and features 12 molecules: AMP, ATP, asparagine (Asn), glutamine (Gln), glutamate (Glu), aspartate (Asp), asparagine synthetase enzyme (ASNe), ASNe complexed with glutamine (ASNe-Gln), ASNe complexed with ammonia (ASNe-NH_3_), β-aspartyl-complex (βAsp-AMP-ASNe-NH_3_), magnesium ions, and ammonia. For clarity, water and pyrophosphate are not shown. Redrawn from [46].

Asparagine is synthesised through the ATP-dependent transfer of the amino group of glutamine to a molecule of aspartate to generate glutamate and asparagine, a reaction catalysed by the enzyme asparagine synthetase. A continuous Petri net model based on mass-action kinetics describing this reaction has been developed (Figure 4) [46] on the basis of biochemical analyses of wheat asparagine synthetases expressed in *E. coli*. Heterologously-expressed maize and soybean enzymes have also been analysed [47,48] but the activity of these enzymes was relatively low compared with the wheat enzymes. The analyses of the wheat enzymes showed that the early stages of the reaction (r1 and r2 in Figure 4) can proceed faster than and independently of the later stages (r3 and r4), consistent with the hypothesis proposed by Gaufichon et al. [49] that steps r1 to r4 occur sequentially rather than simultaneously. Thus, despite the overall equation of the reaction being Glutamine + Aspartate + ATP → Glutamate + Asparagine + AMP + PPi, glutamate synthesis can proceed independently of asparagine synthesis when aspartate is not available.

Wheat contains five asparagine synthetase genes per genome, in four groups, called *TaASN1*, *TaASN2*, *TaASN3.1*, *TaASN3.2*, and *TaASN4* [46,50,51]. This gene family structure is conserved throughout the Triticeae [51]. All of the enzymes have molecular masses between 65 and 67 kDa [46], and the enzymes analysed to model the reaction, TaASN1 and TaASN2, were found to be biochemically very similar [46].

*TaASN1*, *TaASN2*, and *TaASN4* are all single copy genes, located on chromosomes 5, 3, and 4, respectively, of each genome (A, B, and D), although some bread wheat (*Triticum aestivum*) varieties and emmer wheat variety Zavitan (*Triticum dicoccoides*; genomes AABB) lack a group 2 gene in the B genome [46,51]. The two *TaASN3* genes, *TaASN3.1* and *TaASN3.2*, are present on chromosome 1 of each genome [46,51].

Gao, et al. [50] examined the expression of *TaASN1*, *TaASN2*, and *TaASN3* and showed *TaASN2* to be the most highly expressed in the grain, in both the embryo (part of the bran fraction) and endosperm (the white flour fraction). This was confirmed by RNA-seq analysis of gene expression in two genotypes of wheat grown with and without sulphur feeding [52]. The two genotypes were variety Spark and a doubled haploid, SR3, from a Spark × Rialto mapping population [27]. SR3 was selected because it had been shown to have much lower concentrations of free asparagine in its grain than either of its parents (1.68 mmol per kg compared with 2.71 mmol per kg for Spark when grown in compost, for example; a difference of 61% with respect to the lower figure) [27]. This analysis revealed a much higher total asparagine synthetase gene expression in the embryo than in the endosperm (> 10-fold difference), with *TaASN2* being the most highly expressed asparagine synthetase gene in in both tissues, whether the wheat had been supplied with sulphur or not. This was despite the expression of a B genome *TaASN2* homeologue being undetectable [52]. 

**Figure 5:**
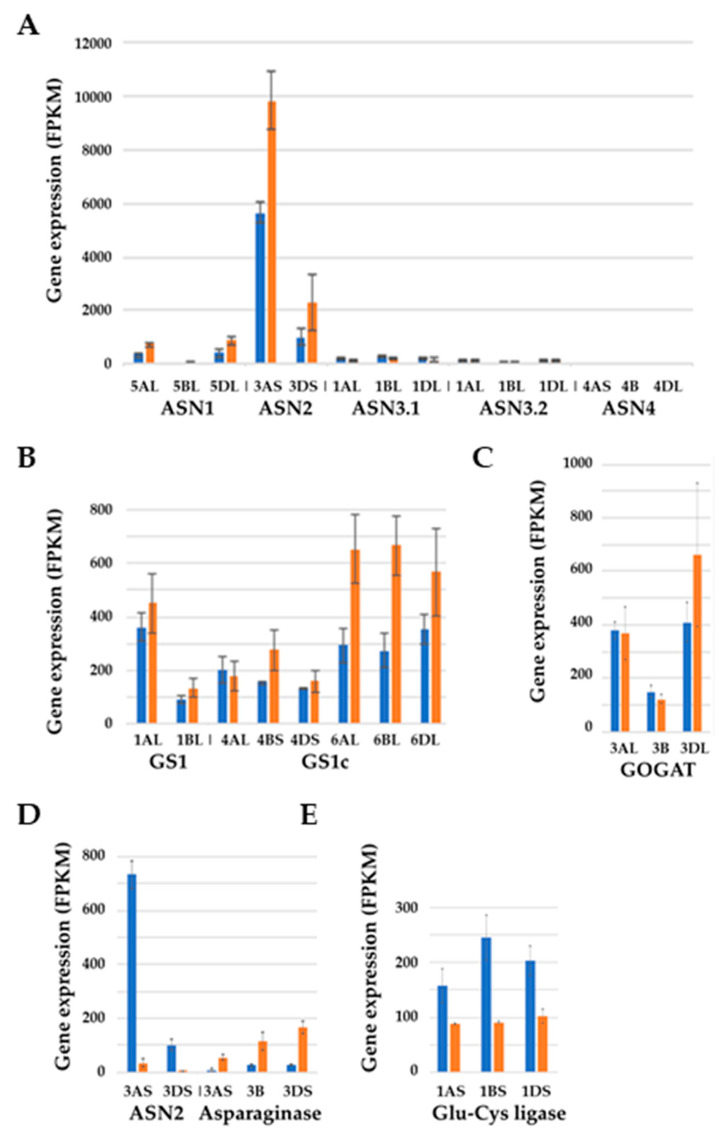
Graphical representation (means and standard errors) showing the effects of sulphur on the expression levels (fragments per kilobase of transcript per million mapped reads (FPKM)) of selected genes in developing grain of wheat (*Triticum aestivum*) genotype SR3 at 21 days post-anthesis. Sulphur was either supplied (blue columns) or withheld (brown columns). Results for each homeologue are shown separately, with chromosomal locations indicated. (**A**) Expression of asparagine synthetase genes *ASN1*, *ASN2*, *ASN3.1*, *ASN3.2*, and *ASN4* in the embryo. The increase in expression of the *ASN1* homeologue on chromosome 5D in response to sulphur deficiency was significant (*p* = 0.0478), as was the increase in expression of both *ASN2* homeologues (*p* = 0.038 and 0.047 for the 3A and 3D homeologues, respectively). (**B**) Expression of glutamine synthetase genes, *GS1*, in the embryo. The increase in expression of the 6AL and 6BL homeologues in response to sulphur deficiency was significant (*p* = 0.0401 and 0.0164, respectively). (**C**) Expression of glutamate synthase (GOGAT) in the embryo. Expression of the D genome gene did increase in response to sulphur deficiency, and although the change was not statistically significant, it followed the trend for *ASN2* and *GS1*. (**D**) Contrasting responses of asparagine synthetase gene *ASN2* and the most highly expressed asparaginase gene in the endosperm. The increase in expression of all three asparaginase homeologues in response to sulphur deficiency was significant ***(****p* < 0.01), as was the decrease in expression of the *ASN2* homeologues (*p* < 0.001 for both). (**E**) Expression of glutamate-cysteine ligase in the endosperm. The reduction in expression of the 1BS and 1DS genes in response to sulphur deficiency was significant (*p* < 0.001 and *p* = 0.028, respectively). Replotted from RNA-seq data provided in [52].

The expression of the asparagine synthetase genes in SR3 in the sulphur-fed and-deprived conditions in the embryo at 21 days post-anthesis (dpa) is shown in Figure 5A. The figure illustrates very clearly that the A genome homeologue of *TaASN2* is much more highly expressed (>3-fold difference) than the D genome homeologue. By this timepoint, the effects of sulphur were becoming evident (much more so than at 14 dpa [52]), with expression of the *TaASN1* homeologue on chromosome 5D and both *TaASN2* homeologues increasing in response to sulphur deficiency. The same response was not observed in Spark, possibly because Spark was behind SR3 developmentally and it was too early to see the response [52]. The opposite occurred in the endosperm at the same timepoint, but with expression so much lower in the endosperm than the embryo that it was concluded that the embryo was the tissue that controlled grain asparagine levels, and that the additional free asparagine that accumulates in the endosperm in response to sulphur deficiency [32] must be imported from the embryo or elsewhere.

Asparagine synthetase is, of course, not the only enzyme that affects asparagine synthesis and breakdown, and an extensive network has been compiled of the genes, enzymes, transcription factors, and regulatory proteins that are likely to be involved [53]. This network was used to filter the RNA-seq data to focus on the genes whose expression was consistent with a role in the changes in free asparagine accumulation observed in response to sulphur availability [52]. The observation that changes in asparagine synthetase gene expression in response to sulphur were much more evident at 21 dpa than earlier also applied to other genes, and the responses were again much more evident in SR3 than Spark [52].

Cytosolic glutamine synthetase (*GS1*) gene expression showed similar responses to asparagine synthetase, with higher expression in the embryo than endosperm, and expression increasing in the embryo of SR3 at 21 dpa in response to sulphur deficiency (Figure 5B) [52]. Glutamine synthetase catalyses the ATP-dependent condensation of glutamate and ammonia to form glutamine [54]. The amido nitrogen of glutamine is then transferred to 2-oxoglutarate to make glutamate by the enzyme glutamate synthase (glutamine oxoglutarate aminotransferase; GOGAT) using NADH/NADPH or ferredoxin as reductants. A gene encoding GOGAT also showed much higher levels of expression in the embryo than the endosperm [52], with a trend for increased expression of one of the homeologues under sulphur deficiency (Figure 5C), although this was not statistically significant [52].

Wheat also has a family of genes encoding asparaginases, enzymes that convert asparagine to aspartate and ammonia, with the ammonia being recycled into amino acid metabolism by glutamine synthetase. We suggest that the fact that wheat grain has asparaginase activity is evidence of the use of free asparagine as a nitrogen storage molecule, with the asparaginase required to remobilize the nitrogen stored as free asparagine. The expression of asparaginase genes in wheat endosperm appears to be consistent with such a role, responding to sulphur in the opposite way to *ASN2* by rising in response to sulphur deficiency while asparagine synthetase gene expression falls (Figure 5D). Note the different scales on the *y*-axis of Figure 5A,D for asparagine synthetase, and the much higher expression of *ASN2* in the embryo than the endosperm [52]. We speculate that asparaginase is expressed in readiness to remobilise the free asparagine if sulphur becomes available, for example, if the roots reach a source of sulphur in the soil, or at germination. This would mean that the enzyme would have to be inactive until required, and more research is required on how asparaginase enzymes might be inactivated and reactivated post-translationally.

Another gene that showed a substantial change in expression was one encoding the enzyme glutamate-cysteine ligase. The expression of this gene in the endosperm decreased substantially in response to sulphur deficiency (Figure 5E). This enzyme, also known as γ-glutamyl cysteine synthetase or GSH1, interacts with asparagine metabolism only in that it utilizes glutamate. However, it is an enzyme that attracts a lot of attention because it catalyses the first and rate-limiting step in the biosynthetic pathway for glutathione (C_10_H_17_N_3_O_6_S), an important, sulphur-containing antioxidant [55]. Reduced expression of this gene might be expected to conserve cysteine when the sulphur required for making it is scarce, but also to reduce a cell’s ability to cope with oxidative stress. Greatly reduced glutathione levels have also been observed in sulphur-deprived rice plants [56].

**Figure 6:**
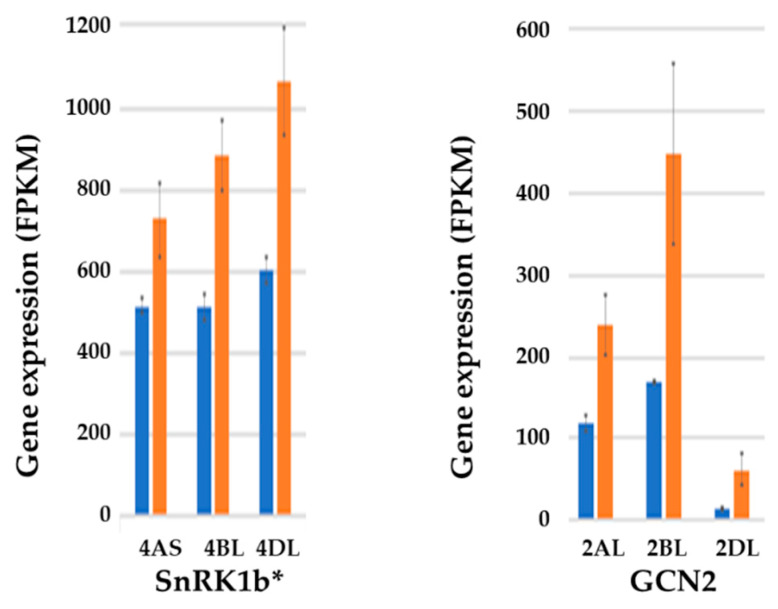
Graphs (means and standard errors) of expression levels (FPKM) of genes encoding SNF1-related protein kinase-1 (SnRK1) type b* (left) and general control nonderepressible-2 (GCN2) in the embryo of wheat (*Triticum aestivum*) genotype SR3 at 21 days post-anthesis. Sulphur was either supplied (blue columns) or withheld (brown columns). Results for each homeologue are shown separately, with chromosomal locations indicated. The increase in expression of the 4BL and 4DL homeologues of the SnRK1b* gene in response to sulphur deficiency was significant (*p* = 0.0426 and *p* = 0.0377, respectively), as was the increase in expression of all three *GCN2* homeologues (*p* = 0.03114, < 0.01, and 0.01313 for the 2AL, 2BL, and 2DL homeologues, respectively). Replotted from RNA-seq data provided in [52].

The RNA-seq analysis also revealed changes in expression of genes encoding regulatory protein kinases, sucrose nonfermenting-1 (SNF1)-related protein kinase-1 (SnRK1) and general control nonderepressible-2 (GCN2), in response to sulphur [52] (Figure 6). Both of these major regulators of metabolism have been implicated in the control of asparagine synthetase gene expression before [57,58], and the expression of one type of *SnRK1* gene (SnRK1b*) and *GCN2* showed responses to sulphur deficiency that would be consistent with such a role (Figure 6), both increasing in expression in the embryo in response to sulphur deficiency.

SnRK1 may act through phosphorylation and regulation of, amongst other things, basic leucine zipper domain (bZIP) transcription factors, and several bZIP transcription factors with SnRK1 target sites were also shown to be expressed in the RNA-seq analysis. Three of these transcription factors, Opaque2/bZIP9, SPA/bZIP25, and BLZ1/OHP1/bZIP63, are known to bind a putative regulatory motif known as the N-motif or GCN4 box [59]. This motif, consensus sequence ATGAGTCAT, is present in the promoter region of wheat *ASN1* [50] and all the cereal *ASN1* genes for which sufficient nucleotide sequence data are available. Intriguingly, this motif is also present in some storage protein genes [60], where it functions to enhance gene expression when sufficient nitrogen is available and repress it when nitrogen is in short supply [61]. Its role in response to sulphur availability has never been investigated. *ASN1* does respond to nitrogen as well as sulphur availability in both bread and pasta wheat [50,62], as well as a range of abiotic stresses, such as salt stress, osmotic stress, and abscisic acid (ABA) [63].

## 7. Effects of Sulphur Nutrition on Genes Involved in Carbon Metabolism

It is possible that SnRK1 responds to sulphur deprivation as a result of down-regulated glucose metabolism, since glucose metabolism is known to decrease during sulphur deficiency in Arabidopsis [39]. The involvement of SnRK1 provides a possible conduit for sulphur to affect carbon metabolism and starch synthesis. For example, SnRK1 over-expression has been shown to increase starch content of potato tubers [64], in part through increased expression of sucrose synthase (SuSy). SuSy catalyses the interconversion of sucrose with UDP-glucose and fructose and, despite its name, the equilibrium of the reaction under physiological conditions is very much in the cleavage direction. The reaction is the first step in the starch biosynthetic pathway, the UDP-glucose being converted to glucose 1-phosphate by UDP-glucose pyrophosphorylase, and glucose 1-phosphate (with ATP) then being converted to ADP-glucose and pyrophosphate by ADP-glucose pyrophosphorylase (AGPase). ADP-glucose is the glucose donor for the elongation of glucan chains by starch synthases. AGPase activity is also modulated by SnRK1 in potato tubers, via gene expression [64] and redox activation [65]. There is also evidence that SnRK1 has a role in starch accumulation in rice and sorghum grain [66,67], and in sorghum and barley pollen [67,68].

**Figure 7:**
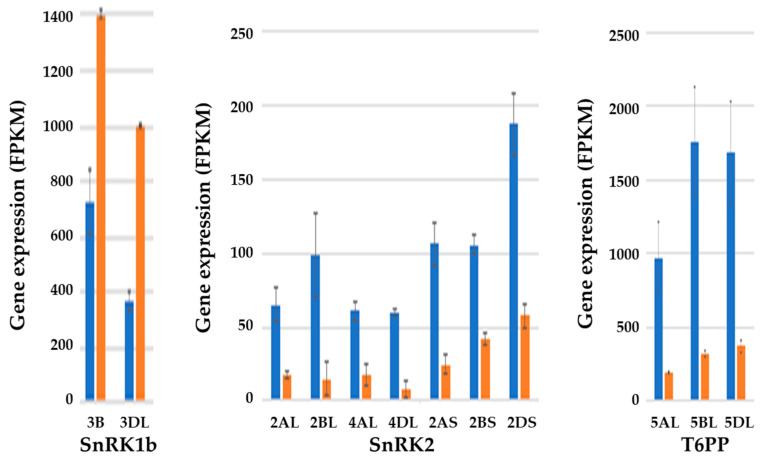
Graphical representation (means and standard errors) showing the effects of sulphur on the expression levels (FPKM) of selected genes in the endosperm of developing grain of wheat (*Triticum aestivum*) genotype SR3 at 21 days post-anthesis. Sulphur was either supplied (blue columns) or withheld (brown columns). Results for each homeologue are shown separately, with chromosomal locations indicated. (**Left panel**) SNF1-related protein kinase-1 (*SnRK1*) type b. The increase in expression of both homeologues in response to sulphur deficiency was significant (*p* < 0.01). (**Middle panel**) *SnRK2*. The decrease in expression of all homeologues of all three genes in response to sulphur deficiency was significant (*p* < 0.01 for 2AL and 2BS; *p* < 0.001 for all others). (**Right panel**) Gene encoding trehalose 6-phosphate (T6P) phosphatase. The decrease in expression of all three homeologues in response to sulphur deficiency was significant (*p* < 0.001). Plotted from RNA-seq data provided in [52].

The RNA-seq analysis of wheat grain produced with and without sulphur supplied [52] revealed that *SnRK1* gene expression increased in the endosperm in response to sulphur deprivation (Figure 7) as well as the embryo (Figure 6), but in the endosperm it was a different class of *SnRK1*, the endosperm-specific *SnRK1b* that is unique to cereals [69]. Plants have two groups of related protein kinases, SnRK2 and SnRK3, and three *SnRK2* genes, on chromosomes 2AL and 2BL, 4AL and 4DL, and 2AS, 2BS, and 2DS, showed marked decreases in gene expression in response to sulphur deprivation (Figure 7). SnRK2s are associated with a variety of abiotic stress responses [70], and are integral to the abscisic acid (ABA) response pathway [71,72]. Indeed, the *SnRK2* gene on the long arm of chromosome 2 has already been described in detail and named pkABA1 [73]. It mediates ABA-induced changes in gene expression in response to cold, dehydration, and osmotic stress [74]. The *SnRK2* gene on the short arm of chromosome 2 has been called osmotic stress/ABA-activated protein kinase-2 (*SAPK2*) and has been shown to confer ABA sensitivity and drought tolerance in rice [75]. ABA itself is a hormone strongly associated with stress responses [76], but also with grain maturation and, in particular, the switch from the grain filling to maturation phases of grain development [77]. ABA has a very different effect on SnRK1, promoting its degradation [78], and the degradation of SnRK1 and activation of SnRK2 could be the trigger that pushes developing grain into the maturation phase. The increased *SnRK1* expression and reduced *SnRK2* expression seen in the endosperm in response to sulphur deficiency, therefore, could indicate that sulphur deficiency prolonged the grain filling phase. It is also notable that in this case, nutritional stress had the opposite effect on *SnRK2* gene expression to other abiotic stresses.

Another relevant gene in this story that showed a marked decrease in expression in the endosperm in the sulphur-deficient condition is trehalose 6-phosphate (T6P) phosphatase (Figure 7). T6P traces cellular sucrose levels and is an inhibitor of SnRK1 [79]. The role of T6P phosphatase in controlling T6P levels has not been investigated, but clearly such a dramatic reduction in its expression could have an effect.

**Figure 8:**
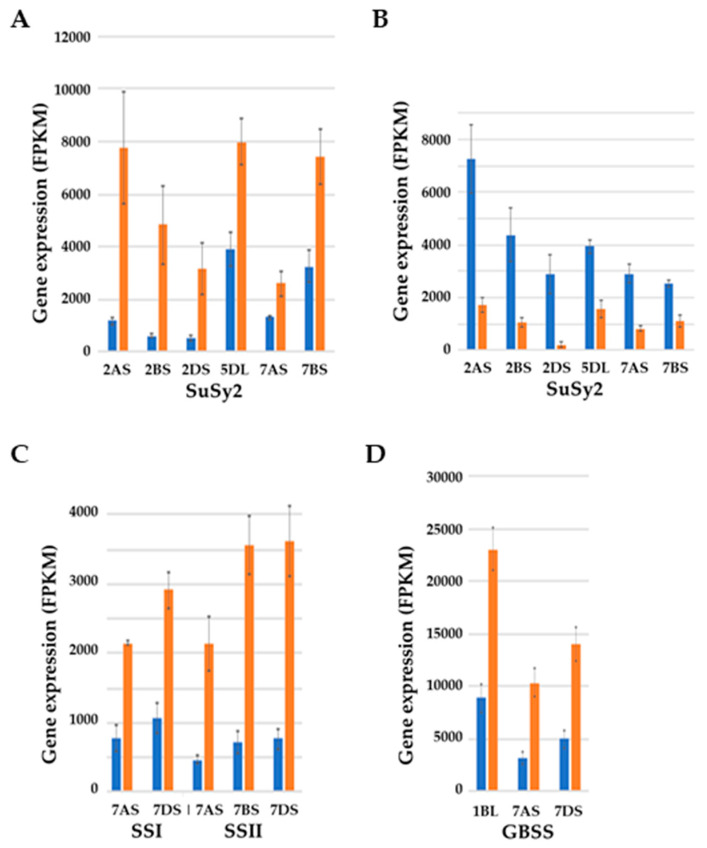
Graphical representation (means and standard errors) showing the effects of sulphur on the expression levels (FPKM) of selected genes in developing grain of wheat (*Triticum aestivum*) genotype SR3 at 21 days post-anthesis. Sulphur was either supplied (blue columns) or withheld (brown columns). Results for each homeologue are shown separately, with chromosomal locations indicated. (**A**,**B**) Sucrose synthase (*SuSy2*) expression in the endosperm and embryo, respectively. The increase in expression in the endosperm in response to sulphur deficiency was significant (*p* < 0.01 for the 5DL, 7AS, and 7BS genes; *p* < 0.001 for the 2AS, 2BS, and 2DS genes), as was the decrease in expression in the embryo (*p* < 0.01 for the 5DL and 7BS genes, *p* < 0.001 for all others). (**C,D**) Soluble and granule-bound starch synthase gene expression, respectively, in the endosperm. The increase in expression in response to sulphur deficiency was significant for all of the genes and homeologues shown (*p* < 0.01 for all *SSI* genes, *p* < 0.001 for all *SSII* genes, *p* < 0.001 for all *GBSS* genes). Plotted from RNA-seq data provided in [52].

There were also substantial increases in *SuSy* and starch synthase gene expression in the endosperm (Figure 8). Two *SuSy* genes were initially characterized in wheat [80], with the *SuSy2* (or *Sus2*) gene expressed predominantly in the endosperm and associated with grain yield [81,82] and the *SuSy1* (*Sus1*) gene expressed in roots and leaves as well as in seeds and induced by anaerobic conditions or cold stress [83]. *SuSy1* and *SuSy2* have been reported to be located on chromosomes 7 and 2, respectively [81,82,84], but genome data suggest that this may be a simplification, with genes annotated as *SuSy1* on chromosomes 3, 4, and 6, as well as 7, and genes annotated as *SuSy2* on chromosomes 1, 4, 5, and 7, as well as 2. In addition there is another gene on chromosome 4 annotated as *SuSy4*. RNA-seq data [52] showed the *SuSy2* genes on chromosomes 2AS, 2BS, 2DS, 5DL, 7BS, and 7DS to be the most highly expressed in both the embryo and endosperm. In the endosperm, expression of these genes increased dramatically in response to sulphur deficiency (Figure 8A), while it decreased in the embryo (Figure 8B) (note that *SnRK1b*, the expression of which increased in response to sulphur deficiency in the endosperm (Figure 7), is not expressed in the embryo and therefore cannot be regulating *SuSy* gene expression in that tissue). 

Starch synthases are also encoded by multigene families and there are both soluble and granule-bound forms of the enzyme (SS and GBSS, respectively) [85]. In the RNA-seq data [52], *SS1* genes were identified on chromosome 7; *SSII* on chromosomes 1, 6, and 7; *GBSSI* genes on chromosomes 1 and 7; and *GBSSII* genes on chromosome 2. However it was the *SS1* and *SSII* genes on chromosome 7A, 7B, and 7D and the *GBSSI* genes on chromosomes 1B, 7A, and 7D that were most highly expressed. In the endosperm, the expression of these genes also increased substantially in response to sulphur deficiency (Figure 8C,D).

Starch and protein content were not compared in the sulphur-fed and –starved grain used for the RNA-seq analysis [52], but clearly the dramatic increase in *SuSy2* and starch synthase gene expression suggests that the starch biosynthetic pathway was upregulated in response to sulphur deficiency. The increase in *SnRK1b* expression would be consistent with that metabolic regulator playing a role in channelling more carbon through the starch biosynthetic pathway, possibly in response to the ability to make proteins being compromised and more carbon being available for starch instead. If this is correct, it would indicate that different forms of SnRK1 were involved in regulating accumulation of asparagine in the embryo (SnRK1b*) and starch in the endosperm (SnRK1b) in response to sulphur availability.

## 8. Conclusions

It is not surprising that plant nutrition can have profound effects on the composition of cereal grains and other crop storage organs, and that this in turn can affect the nutritional value and processing properties of the crop. Indeed, the effect of sulphur availability on yield and protein content of wheat grain has been the subject of extensive study for several decades (see [2], for example). However, it was perhaps less predictable that some of the nitrogen that is normally stored as protein in wheat grains should instead be stored as free asparagine when sulphur supply to the plant is inadequate. It was an even greater shock to the food industry and its supply chain when it was discovered that this free asparagine could be converted to acrylamide, a highly undesirable contaminant, during everyday cooking and processing, leading to a major food safety and regulatory compliance problem.

In this review we have described the predominant route for that conversion in the Maillard reaction. We have reviewed the evidence for the dramatic increase in free asparagine accumulation in response to sulphur deficiency in wheat, and how sulphur and nitrogen have interacting effects. We reiterate our advice that sulphur fertilizer applied to wheat destined for human consumption should be applied at a rate of 20 kg (50 kg SO_3_ equivalent) per hectare, and that nitrogen fertilizer should be accompanied with sulphur. We have described the changes in gene expression that underlie the accumulation of free asparagine in response to sulphur deficiency. Asparagine synthetase-2 (*ASN2*) gene expression in the embryo plays a key role in the response, with regulatory protein kinases SnRK1b* and GCN2 implicated in its regulation and several bZIP transcription factors possibly involved. Clearly more work needs to be done to elucidate that signalling pathway in its entirety and show how it is integrated with other signalling networks. Free asparagine levels also increase in response to pathogen infection, for example [36,86], and SnRK1 is also implicated in that response [87]. Genes encoding glutamine synthetase and glutamate synthetase (GOGAT), as well as asparaginase, also respond to sulphur deficiency, but we do not know if they are regulated by the same signalling pathways. Reviewing RNA-seq data on the effect of sulphur deficiency also revealed for the first time a large reduction in expression of glutamate-cysteine ligase in the endosperm, suggesting that the ability to synthesise the important sulphur-containing antioxidant, glutathione, could be compromised.

The review of RNA-seq data also showed for the first time unexpected effects on carbon metabolism. Notably, both sucrose synthase and starch synthase gene expression increased substantially in the endosperm in response to sulphur deficiency, as did expression of a different form of SnRK1, SnRK1b. SnRK1 has been shown previously to play a role in controlling sucrose synthase gene expression in potato tubers and has been associated with starch synthesis in cereal grains [64,66,67]. This suggests that the capacity to synthesise starch could be upregulated in response to sulphur deficiency to store the carbon that is not being used to make proteins.

The notion that sulphur, carbon, and nitrogen metabolism could be integrated is not new; indeed, it has been the focus of many studies. However, these have predominantly focused on the convergence of these three hugely important metabolic systems on cysteine or methionine (see [88], for example) or on effects of sulphur deprivation on photosynthesis (see [54], for example). The apparent overlap between the regulation of free asparagine accumulation and starch biosynthesis in response to sulphur in wheat grain is therefore unexpected. The review of these RNA-seq data also re-emphasised the complexity of these sorts of analyses, with differential effects of sulphur on different members of multigene families and even different homeologues of the same gene.

The overall conclusion that we draw from this review is that crop nutrition may have profound and sometimes unexpected effects on crop composition, with implications not only for the nutritional and processing properties of the crop but also for food safety.
